# Seasonal variation in salivary cortisol but not symptoms of depression and trait anxiety in pregnant women undergoing an elective caesarean section

**DOI:** 10.1016/j.psyneuen.2019.05.029

**Published:** 2019-10

**Authors:** Samantha M. Garay, Katrina A. Savory, Lorna A. Sumption, Richard J.A. Penketh, Ian R. Jones, Anna B. Janssen, Rosalind M. John

**Affiliations:** a Biomedicine Division, School of Biosciences, Cardiff University, Cardiff, CF10 3AX, United Kingdom; b Department of Obstetrics and Gynaecology, University Hospital Wales, Cardiff, Wales, CF144XW, United Kingdom; c National Centre for Mental Health, MRC Centre for Neuropsychiatric Genetics and Genomics, School of Medicine, Cardiff University, Cardiff, Wales, CF14 4XN, United Kingdom

**Keywords:** Seasons, Maternal salivary cortisol, Maternal mood disorders, Custom birthweight centiles, Placental weight

## Abstract

**Objectives:**

Seasonal changes in mood and behaviour are commonly reported in the general population but considerably less is known regarding seasonality and pregnancy. This study investigated the relationship between seasons and depression and anxiety symptoms, salivary cortisol concentrations, custom birthweight centiles (CBWC) and placenta weight for pregnant women living in South Wales.

**Methods:**

This study utilised data from the longitudinal Grown in Wales (GiW) cohort. Women were recruited at the presurgical elective caesarean section (ELCS) appointment, when they provided saliva samples and completed the Edinburgh Postnatal Depression Scale (EPDS) and trait subscale of the State-Trait Anxiety Inventory (STAI). Data on birthweight and placental weight was extracted from medical notes. Seasonal data was available for 316 participants.

**Results:**

No association was identified between seasons and EPDS (*p* = .178), STAI scores (*p* = .544), CBWC (*p* = .683) or placental weight (*p* = .857). Significance was identified between seasons and salivary cortisol concentration (p<.001), with highest levels in autumn and winter. Adjusted linear regression identified spring (B=−.05, *p=*.007, 95% CI −.09, −.01) and summer (B=−.06, *p* = .001, 95% CI −09, −.02) compared to autumn, and spring (B=−.05, *p=*.009, 95% CI −.09, −.01) and summer (B=−.06, *p=*.002, 95% CI −.10, −.02) compared to winter to be associated with decreased cortisol concentrations.

**Conclusion:**

This study found no association between season and maternally-reported mental health symptoms, birthweight by CBWC or placental weight but did between season and term salivary cortisol. This finding will have implications for studies that do not account for seasonality when using salivary cortisol as a biomarker.

## Highlights

• Few studies take into account the impact of season on pregnancy cohort data.
• No evidence of seasonality in customised birth weight or placental weight.
• No evidence of seasonality in maternally reported symptoms of anxiety/depression.
• Season of birth linked to term maternal salivary cortisol levels.
• Maternal cortisol higher in autumn/winter compared to spring/summer.

## 1 Introduction

The Developmental Origins of Health and Disease (DOHaD) hypothesis postulates that adverse environmental exposures during critical periods of intrauterine development lead to changes in the fetus that persist after birth impacting the individual’s genetically determined health trajectory. These changes increase the risk of developing diseases later in life such as type 2 diabetes, hypertension, heart disease and psychiatric disorders (Barker, 1990; Gluckman and Hanson, 2004; O’Donnell et al., 2014; Van den Bergh et al., 2017). Adverse exposures can include poor maternal diet and smoking as well as maternal mood disorders such as prenatal depression or anxiety. One relatively unexplored area of research is the impact of seasons on maternal mood and, consequently, subsequent outcomes for the child.

Seasonal affective disorder (SAD) is the recurrence of major depression symptoms in winter (Melrose, 2015). Seasonal variation in mood has also been identified in the general population. In the United States Harmatz et al. (2000) identified highest Beck Depression Inventory (BDI) scores in winter and lowest in summer, with females showing stronger seasonal variation. In Norway Stordal et al. (2008) identified the highest prevalence of comorbid anxiety and depression in spring and October. Research findings on the relationship between seasons and maternally reported anxiety symptoms are conflicting, with a focus largely on postpartum depression (Jewell et al., 2010; Panthangi et al., 2009; Sit et al., 2011; Sylvén et al., 2011), while a recent large study in Sweden found no consistent seasonal patterns in peripartum depressive symptoms (Henriksson et al., 2017).

Cortisol measurements are utilised widely as a robust biomarker in stress related research (Hellhammer et al., 2009) including mental health issues such as maternal anxiety (O’Connor et al., 2014) and depression (Evans et al., 2008). Cortisol is produced by the adrenal cortex and released into the bloodstream in response to signals from the hypothalamus via the pituitary gland, with a well-established diurnal cycle of secretion (Miller et al., 2016). In pregnancy, maternal cortisol levels are known to naturally increase dramatically in response to increasing hypothalamic-pituitary-adrenal (HPA) axis activity, with a return to pre-pregnancy levels postpartum (Conde and Figueiredo, 2014; Obel et al., 2005; Smy et al., 2016). Maternal symptoms of depression and anxiety in pregnancy have been linked to further elevation in maternal cortisol levels leading to the proposal that cortisol mediates the relationship between prenatal stress and postnatal outcomes (Glover, 1999). In the general population various factors have been suggested to influence cortisol levels including age and gender (Miller et al., 2016; Roelfsema et al., 2017), with highly mixed findings regarding the seasons associated with highest and lowest cortisol levels (Fischer et al., 2017; Hadlow et al., 2014; King et al., 2000; Miller et al., 2016; Persson et al., 2008). Although research in the general population may not be relatable to pregnancy due to this up-regulation of the HPA axis, both fetal sex (Janssen et al., 2018) and parity (Gillespie et al., 2018) are associated with maternal cortisol. Research on seasonal variation in cortisol levels in pregnancy is limited to one study that utilised hair cortisol concentrations as a proxy for exposure during the third trimester of pregnancy and reported concentrations to be higher overall in summer and autumn (Braig et al., 2015).

Understanding the causes of mood disorders in pregnancy is important because these are linked to an increased risk of low birthweight (Evans et al., 2007), low placental weight, and poorer outcomes for both the infant (Aiken, 2017; Flamant and Gascoin, 2013; Morken et al., 2014) and the mother (Eskild, 2018; Ngo et al., 2015) in the longer term. Several studies have reported seasonal differences in birthweight (Heinonen et al., 2001), although evidence regarding specific seasons and birthweight is mixed (Day et al., 2015; Flouris et al., 2009; Lawlor et al., 2005; Lei et al., 2016; Murray et al., 2000; Poeran et al., 2016; Strand et al., 2011). Despite the importance of these relationships between maternal mood and infant outcomes, there is a lack of research investigating seasonality utilising standardised custom birthweight centiles (CBWC), which are a more accurate system for classifying birthweight by adjusting for various maternal and infant characteristics, overcoming the limitations associated with utilising traditional birthweight classifications (Gardosi, 2012; Gardosi and Francis, 2009; Zhang et al., 2010).

Given the importance of the environment during pregnancy for fetal development and future outcomes, it is vital to understand the potential influence of seasons on the mother’s mood and outcomes for her children. In light of the highly mixed findings in the general population and the paucity of research in pregnancy, this paper investigates the exploratory hypothesis that maternal symptoms of depression and anxiety are influenced by season impacting maternal cortisol, a factor proposed to mediate the relationship between maternal mood and infant outcomes. We tested this hypothesis using data from a cohort of women delivering by booked elective caesarean section (ELCS) in Wales.

## 2 Method

Full ethical approval for the Grown in Wales (GiW) study was obtained via the Wales Research Ethics Committee REC reference 15/WA/0004 and research was carried in accordance with the principles of the Declaration of Helsinki as revised in 2008. The cohort has previously been described in detail in Janssen et al. (2018). Briefly, the GiW study is a longitudinal cohort study in the South Wales region of the United Kingdom that recruited and obtained written consent from term women (37–42 weeks of pregnancy) at their presurgical appointment prior to a booked ELCS, at the University Hospital of Wales (UHW) between 1st September 2015 and 31st November 2016. Women aged 18–45 were invited to participate in the study if it was a singleton pregnancy without fetal anomalies and infectious diseases.

### 2.1 Participants

Initially, 355 women were recruited to the GiW study, with seven later withdrawing. Seasonal data was available for 347 women. This study focused on the 316 Caucasian participants to minimise the confounder of ethnicity which can impact cortisol (Urizar Jr. et al., 2018).

### 2.2 Materials

Data was gathered at the presurgical appointment prior to a booked ELCS and immediately after birth, through an extensive questionnaire and notes recorded by the research midwife. Northern Meteorological Seasons were utilised in this analysis, defined as spring (March 1st to May 31st), summer (June 1st to August 31st), autumn (September 1st to November 31st) and winter (December 1st to February 28th/29th).

#### 2.2.1 Maternal depression and anxiety symptoms

Maternal depression symptoms were assessed utilising the Edinburgh Postnatal Depression Scale (EPDS) (Cox et al., 1987). This has been validated for use in the antenatal period, with a review identifying sensitivity and specificity estimates ranging from 64 to 100% and 73–100% respectively (Kozinszky and Dudas, 2015). The EPDS is a 10-item questionnaire in which participants are required to select an answer from 4 possible responses that comes closest to how they have felt in the past 7 days. The maximum possible score on the EPDS is 30, with a score of ≥13 indicative of probable depression (Cox et al., 1987; Matthey et al., 2006).

Trait anxiety was assessed using a subscale from the Spielberger State-Trait Anxiety Inventory (STAI) (Spielberger et al., 1983), which has been validated for use in pregnancy (Meades and Ayers, 2011), with Grant et al. (2008) reporting sensitivity and specificity of 80.95% and 79.75% respectively. The trait subscale of the STAI is a 20-item questionnaire that assesses anxiety levels in general, with all items rated on a 4-point scale (*e.g*. from “Almost never” to “Almost always”). The maximum possible score on the trait subscale is 80, with a score of ≥40 recommended as indicative of high anxiety (Barnett and Parker, 1985).

#### 2.2.2 Maternal cortisol

Cortisol was derived from maternal saliva samples. Participants provided one sample of saliva on the day of recruitment at least 30 min after their last meal, with >90% of the samples collected between 10 a.m. and 1 pm. Saliva was collected in Sarstedt salivettes under the supervision of the research midwife consenting the participant. The participant was asked to place the swab in the mouth and chew for about 60 s, and return the swab to the plastic tube with the midwife noting the time and date that the sample was taken. Samples were kept at −80 °C until cortisol concentration (μg/dL) was determined in duplicate repeats at Anglia Ruskin University by the Human Tissue Authority licenced Salimetrics.

#### 2.2.3 Maternal characteristics and lifestyle

Data on maternal demographics and lifestyle was collected from the extensive questionnaire completed on recruitment to the study. Demographic data included ethnicity, income and education. Lifestyle data included information on smoking, alcohol intake and exercise. Parity, maternal age at booking and maternal weight to calculate gestational weight gain were taken from notes recorded by the research midwife. Welsh Index of Multiple Deprivation (WIMD) 2014 scores were calculated from anonymised postcodes (http://wimd.wales.gov.uk).

#### 2.2.4 Birth outcome measures

Placental weight and infant gender were taken from notes recorded by the research midwife. CBWC were calculated via the GROW bulk centile calculator (UK) (Gardosi and Francis, 2016), utilising data collected from the questionnaire and midwife notes on maternal height, weight, ethnicity and parity as well as infant birthweight, gender and gestational age.

### 2.3 Statistical analysis

All analyses were undertaken utilising IBM SPSS Statistics Version 25. Cortisol data was assessed for outliers, which were defined as values more than 2 standard deviations away from the mean and confirmed by the visual inspection of the histogram, normal Q-Q plot and box and whisker plot. Analysis was run both with and without outliers and no statistically significant differences were identified. Consequently the cortisol outliers were removed from the analysis. After assessing for normality, via consideration of skewness and kurtosis, Shapiro-Wilk significance and normality plots, cortisol concentration was judged to be parametric, whilst EPDS and STAI scores, CBWC and placental weight were non-parametric. One-way ANOVA with Tukey’s post-hoc analysis was employed to investigate the association between seasons and salivary cortisol concentration, whilst Kruskal-Wallis H test was utilised for EPDS and STAI, CBWC and placental weight. To further investigate the relationship between seasons and cortisol both unadjusted and adjusted multiple linear regression was utilised, with autumn and winter as reference categories in separate models. The analysis was adjusted for time point of cortisol sample collection and potential confounding variables identified from existing literature. These variables were fetal sex, parity, maternal age, maternal body mass index (BMI) at booking, exercise, alcohol intake and smoking in pregnancy. Linear regression was also applied to assess firstly the relationship between cortisol and the birth outcomes of CBWC and placental weight, and secondly the influence of seasonality of cortisol in this relationship, by separating the analysis by season.

## 3 Results

Demographic data for the 316 participants for which seasonal data was available is displayed in Table 1. Of these, data was available for EPDS (n = 310), STAI (n = 310), CBWC (n = 313), placental weight (n = 301) and cortisol (n = 284). Table 2 displays the median and interquartile range (IQR) or mean and standard deviation (SD) values for each variable by season.

**Table 1:** Demographics for the 316 participants for which seasonal data was available.

Demographics	% (n) or median (IQR)
Maternal age at booking	33 (6.00)
Parity, *% (n)*	
Multiparous	79.70 (252)
Nulliparous	20.30 (64)
Gestational weight gain (kg)	14.84 (8.00)
Fetal sex, *% (n)*	
Female	55.00 (172)
Male	45.00 (141)
Highest education level, *% (n)*	
Left before GCSE	6.30 (19)
GCSE & Vocational	24.80 (75)
A-level	12.60 (38)
University	30.80 (93)
Postgraduate	25.50 (77)
Family income, *% (n)*	
<18,000	8.80 (27)
18 – 25,000	9.10 (28)
25–43,000	20.10 (62)
>43,000	49.40 (152)
Do not wish to say	12.70 (39)
Smoking in pregnancy^a^, *% (n)*	
No	89.10 (278)
Yes	10.90 (34)
Alcohol in pregnancy^a^, *% (n)*	
No	65.20 (204)
Yes	34.80 (109)
Strenuous exercise, *% (n)*	
No	83.00 (259)
Yes	17.00 (53)
WIMD score	1212 (1250.50)

BMI, Body Mass Index; WIMD score, Welsh Index of Multiple Deprivation score.

^b^WIMD score has a possible range of 1–1909, with a low score indicative of an area of higher deprivation and conversely a high score indicative of an area of lower deprivation.

a At any point in pregnancy.

**Table 2:** Median (IQR) or mean (SD) for variables of interest in relation to season.

Season	CBWC		Placental weight (kg)		EPDS		STAI		Cortisol (μg/dL)	
	N	Median (IQR)	N	Median (IQR)	N	Median (IQR)	N	Median (IQR)	N	Mean (SD)
Spring	57	52.60 (50.50)	55	.66 (.21)	57	7.00 (5.50)	57	35.00 (12.00)	49	.26 (.10)
Summer	73	56.80 (54.40)	71	.66 (.13)	76	8.00 (5.00)	76	34.50 (10.75)	68	.26 (.09)
Autumn	111	64.30 (51.00)	105	.66 (.18)	108	6.00 (6.00)	107	32.00 (12.00)	104	.32 (.10)
Winter	72	53.65 (44.15)	70	.65 (.17)	69	7.00 (6.50)	70	34.00 (15.00)	63	.31 (.10)
Total	313	57.40 (50.25)	301	.66 (.18)	310	7.00 (6.00)	310	34.00 (12.00)	284	.29 (.10)

EPDS; Edinburgh Postnatal Depression Scale, STAI; State-Trait Anxiety Inventory, CBWC; custom birthweight centile.

**Fig. 1:**
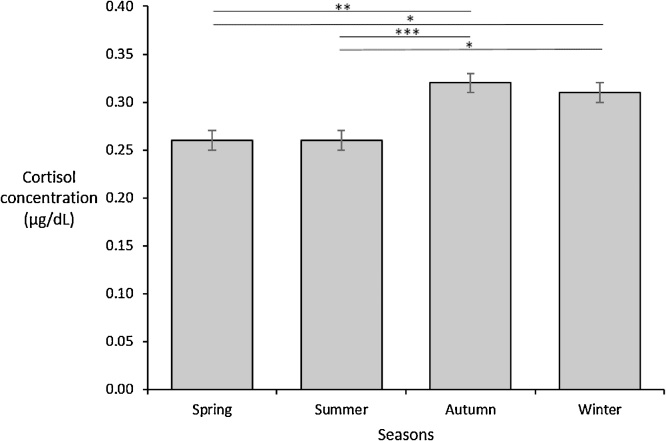
Mean salivary cortisol concentration by season, with error bars representing standard error. * < 0.05 ** < .005 ***< .001.

One-way ANOVA and Kruskal-Wallis H analyses were undertaken to assess the associations between seasons and each variable of interest. No significant associations were identified between seasons and EPDS scores (*p* =  .178), STAI scores (*p* =  .544), CBWC (*p* =  .683) or placental weight (*p* =  .857). However, significance was identified between seasons and cortisol concentration with *F(*3,280*) =* 7.70*, p* <.001. Tukey’s post-hoc analysis further identified significant differences in cortisol concentrations between spring-autumn (mean difference = −.06, *p* =  .002, 95% CI −.11, −.02), spring-winter (mean difference = −.06, *p* =  .018, 95% CI = −.10, −.01), summer-autumn (mean difference = −.06, *p* =  .001, 95% CI −.10, −.02) and summer-winter (mean difference = −.05, *p* =  .016, 95% CI = −.10, −.01). This relationship can also be seen in Fig. 1.

To further investigate the relationship between season and cortisol concentration, multiple linear regression was utilised. Unadjusted, there was a significant association for spring (*p* =  .001) and summer (*p* <  .001) and cortisol concentrations, when compared to autumn (Table 3). There was also a significant unadjusted association for spring (*p=*.004) and summer (*p=*.003) and cortisol concentrations, when compared to winter. These significant associations remained when adjusted for potential confounders; time point of saliva sample collection, fetal sex, parity, maternal age, maternal BMI at booking, exercise, alcohol intake and smoking in pregnancy. Specifically, when adjusted and utilising autumn as the reference, seasons can significantly predict cortisol concentrations as *F*(11,233) = 3.90, *p* <  .001, with spring and summer compared to autumn associated with a decrease in cortisol concentrations of .05 μg/dL and .06 μg/dL respectively (Table 3). Similarly, the model is significant when adjusted and utilising winter as a reference *F*(11,233) = 3.90, *p* <  .001, with spring and summer compared to winter again associated with a decrease in cortisol concentrations of .05 μg/dL and .06 μg/dL respectively (Table 3).

**Table 3:** Multiple linear regression models assessing the association between season and cortisol concentration (μg/dL).

Model	Season	*P*	B	95% CI
Unadjusted	Spring	**.001**	−.06	−.10, -.03
	Summer	**<.001**	−.06	−.09, -.03
	Autumn	*ref*		
	Winter	.740	−.01	−.04, .03

Adjusted^a^	Spring	**.007**	−.05	−.09, -.01
	Summer	**.001**	−.06	−.09, -.02
	Autumn	*ref*		
	Winter	.874	.00	−.03, .04

Unadjusted	Spring	**.004**	−.06	−.09, -.02
	Summer	**.003**	−.05	−.09, -.02
	Autumn	.740	.01	−.03, .04
	Winter	*ref*		

Adjusted^a^	Spring	**.009**	−.05	−.09, -.01
	Summer	**.002**	−.06	−.10, -.02
	Autumn	.874	.00	−.04, .03
	Winter	*ref*		

Ref, reference category.

a Adjusted for time point of cortisol sample collection, fetal sex, parity, maternal age, maternal BMI at booking, exercise, alcohol intake and smoking in pregnancy.

Additionally linear regression was utilised to assess the relationship between salivary cortisol concentration and birth outcomes. There was no significant association identified between cortisol concentration and CBWC (*p* =  .280) or placental weight (*p* =  .446). This analysis was then separated by season to investigate any influence of seasonality of cortisol on this relationship. There remained no association between cortisol and CBWC in spring (*p* =  .437), summer (*p* =  .472), autumn (*p* =  .111) or winter (*p* =  .888), or between cortisol and placental weight in spring (*p* =  .459), summer (*p* =  .379), autumn (*p* =  .242) or winter (*p* =  .933).

## 4 Discussion

Previous studies suggest that maternal cortisol may mediate the reported association between maternal symptoms of anxiety and depression in pregnancy and poorer outcomes for children including lower birthweight. We previously reported that maternal salivary cortisol measured at the presurgical appointment prior to a planned surgical delivery was not associated with maternal EPDS or STAI scores (Janssen et al., 2018). The current study asked whether seasonality, an environmental factor linked to depressed mood in the general population, might contribute to this relationship. We found no association between season of birth and maternal symptoms of anxiety or depression, or the outcomes of birthweight or placental weight. However, we did find a significant association between season of delivery and maternal salivary cortisol. Specifically, the highest levels were identified in autumn and winter, with spring and summer associated with a 0.05 μg/dL and 0.06 μg/dL decrease in cortisol concentration respectively compared to both autumn and winter separately, after adjustment for various potential confounders.

This is the first study to report an effect of seasonality on maternal salivary cortisol concentrations late in pregnancy. We found the highest levels of salivary cortisol in the autumn and winter. Braig et al. (2015), who used hair cortisol measurements, reported the highest concentrations in summer and autumn and lowest in winter. Differences between our study and Braig et al. (2015) may be due to the samples tested (hair versus saliva), population demographics and/or differences in temperature (Flouris et al., 2009) and light (Hadlow et al., 2014), with humidity also suggested to influence hair cortisol levels (Boesch et al., 2015). Despite these differences in findings, our study has important implications. Firstly, salivary cortisol is sensitive to the seasonal changes that occur in the UK, with the limitations discussed later. Secondly, this relationship may offer an explanation for research demonstrating that season of birth influences the risk of developing other conditions later in life (Disanto et al., 2012). For example, schizophrenia prevalence is highest in those born in January and February and these infants are exposed *in utero* to the highest levels of cortisol due to a combination of the pregnancy related increase in cortisol combined with a seasonal increase. Thirdly, our finding has implications for any research investigating cortisol levels in pregnancy. Few studies take seasonality into account which may explain why studies differ in their findings. It should be possible, in light of our results, to reanalyse data from much larger cohort studies to validate our original finding.

Despite cortisol being a widely utilised biomarker in stress research and this study identifying seasonality in salivary cortisol concentrations, we found no relationship between seasons and self-reported depression and anxiety symptoms in our population. Cortisol levels in maternal saliva and the reported maternal mood symptoms of anxiety and depression in our cohort were also not associated (Janssen et al., 2018) indicating that the relationship between maternal mood and maternal cortisol is complex. We are not aware of any existing research investigating seasonality and anxiety symptoms in pregnancy, whilst studies on depression have largely focused only on the postnatal period, with highly variable findings. Sit et al. (2011) and Sylvén et al. (2011) found an association between seasons and postnatal depression, however Jewell et al. (2010) and Panthangi et al. (2009) in the postnatal period and Henriksson et al. (2017) in the perinatal period did not report an association, similar to our findings in pregnancy. Again it is possible these mixed findings are due to variability in methodologies and seasonal extremes in light and temperature. However, it is also noteworthy that our study and many others utilised self-report measures of depression and anxiety symptoms which may have influenced findings, as self-report measures are potentially subject to bias due to socially desirable responding. For example Schoch-Ruppen et al. (2018) identified different associations to birth outcomes when utilising self-report and implicit assessment of maternal mood. As such, whilst the EPDS and STAI are well-established and validated measures it will be important to investigate the association utilising non-self-report measures.

Our study identified no effect of season on CBWC (*i.e*. birthweight) or placental weight. Previous studies have reported highest birthweight in summer and lowest in winter (Day et al., 2015), highest in autumn and lowest in winter (Lawlor et al., 2005), lowest in spring and summer (Murray et al., 2000), highest in autumn and winter (Flouris et al., 2009), highest in spring and winter (Lei et al., 2016), lowest in summer (Poeran et al., 2016), with one review confirming mixed findings across studies (Strand et al., 2011). These mixed findings in birthweight may be explained by variation in methodologies, definition of seasons, sample sizes and sources of cortisol. It is also possible that our use of CBWC, a strength of this study, provides an explanation for the differences in findings. CBWC provide a more accurate system for classifying birthweight, by accounting for various maternal and infant characteristics (Gardosi, 2012; Gardosi and Francis, 2009; Zhang et al., 2010), and have been recommended for use in the UK by the Royal College of Obstetricians and Gynaecologists since 2002 (Gynaecologists, 2013). Despite this we are not aware of any research on seasonality and birthweight utilising customised measures. Moreover, as variation in temperature (Flouris et al., 2009) and light (Hadlow et al., 2014) have been suggested as mechanisms driving this seasonal effect, studies conducted in countries with varying extremes between seasons may differ in findings. However, there is variability even within UK studies which are exposed to similar differences between seasons, and instead may be partly explained by the dates in which data was collected. In existing studies (Day et al., 2015; Lawlor et al., 2005; Murray et al., 2000), birthweight data reflects births from the 1930′s to the 1980′s. Conditions have changed greatly since this time with improvements in healthcare, socioeconomic conditions and lifestyle, known to influence birthweight. We did not identify an association between season and CBWC when utilising birthweight data from 2015–2016, whereas some studies using older data do report an association. One explanation is that the seasonal effect on birthweight is disappearing, perhaps due to improvements in maternal lifestyle, as suggested by Sohn (2016), the first study to systematically investigate seasonality in birthweight over time, and consistent with Jensen et al. (2015) which reported changes in seasonal trends occur over time.

One limitation of our study is the size of the sample. Some studies in this area have larger sample sizes, although the current study is comparable to many studies investigating seasonality and cortisol (Fischer et al., 2017; King et al., 2000; Persson et al., 2008). A second limitation is our focus on a pregnant population of women recruited at the presurgical appointment prior to an ELCS, with singleton pregnancies and no known infection or fetal anomaly. This population is important to study as the global incidence of caesarean sections (CS) has nearly doubled in recent years (Betrán et al., 2016; Boerma et al., 2018), and in Wales alone in 2015–2016 there were 30,254 deliveries of which 26% were by CS with 11.8% being ELCS (Government, 2017). This focus is both a strength and a limitation of the study. The strength lies in that all the women were recruited at the same hospital by two trained research midwives through a single, defined process prior to the ELCS *i.e*. all women were in the same state and highly comparable. For all participants, saliva was collected at recruitment which, for >90% of the participants, was between 10 a.m. and 1 pm at the presurgical appointment prior to an ELCS excluding any systematic differences across the seasons. However, we cannot conclude that our findings apply to pregnant women delivering by other modes. Additionally, salivary cortisol was assayed at a single time point. Ideally repeated measures of cortisol through pregnancy taking into account the cortisol awakening response are required to address this limitation. As a final point, the GiW cohort includes very few pregnancies of non-Caucasian ethnicity and these were excluded from the current study. We chose this approach to minimise the heterogeneity that multiple ethnicities can introduce obscuring significant associations present within different populations (Bornstein et al., 2013). However, while this approach of using very strict population criteria overcomes some of the limitations of a small study size, our findings may not be applicable to other ethnicities living in Wales.

## 5 Conclusion

In this study we explored the impact of season on a number of measures including prenatal depression, prenatal anxiety, maternal cortisol, birthweight and placental weight which have been associated with poorer outcomes in offspring to ascertain whether seasonality might be an important factor to consider when interpreting findings from different studies. We used custom birthweight centiles (CBWC) as the most accurate measurement of infant weight as these account for various maternal and infant characteristics. This study found no association between season and maternal mood symptoms, birthweight or placental weight but did find an association between season and maternal salivary cortisol at term. Our finding that maternal salivary cortisol levels at term were lowest in spring and summer and highest in autumn and winter will certainly have implications for the interpretation of studies that do not take into account seasonality when using salivary cortisol as a biomarker in pregnancy. Moreover, with the suggestion of seasonality and risk of psychiatric disorders, this is a particular association that would benefit from further study.

## Author contributions

SMG, ABJ and RMJ: Conceptualization; SMG, KS and LS: Data curation; SMG: Formal analysis; RMJ, RJAP and IRJ: Funding acquisition; SMG and RMJ: Investigation; SMG, ABJ, RJAP and RMJ: Methodology; RMJ: Project administration; RMJ: Supervision; SMG: Validation; SMG: Roles/Writing – original draft; RMJ: Writing – review & editing.

## Funding

SMG was supported by a GW4 BioMed MRC DTP PhD studentship (MR/N013794/1). KAS was supported by The Waterloo Foundation (918–3022). LS was supported by GW4 SWBio BBSRC DTP PhD studentship (BB/M009122/1). The Grown in Wales study was funded by MRC grant (MR/M013960/1).

## Conflict of interest statement

SMG, KAS, LS, RJAP, IRJ, ABJ and RMJ declare that the research was conducted in the absence of any commercial or financial relationships that could be construed as a potential conflict of interest.

## Declarations of interest

None.
